# Decompression for lumbar spinal stenosis at the intrathecal catheter insertion site during intrathecal baclofen therapy: a case report

**DOI:** 10.1186/s13256-023-03959-1

**Published:** 2023-06-11

**Authors:** Yasutaka Takagi, Hiroshi Yamada, Hidehumi Ebara, Hiroyuki Hayashi, Hiroyuki Inatani, Kazu Toyooka, Akari Mori, Yoshiyuki Kitano, Aki Nakanami, Kenji Kagechika, Tetsutaro Yahata, Hiroyuki Tsuchiya

**Affiliations:** 1 Department of Orthopaedic Surgery, Tonami General Hospital, 1-61 Shintomi-cho, Tonami City, Toyama 939-1395 Japan; 2 Department of Rehabilitation Medicine, Tonami General Hospital, 1-61 Shintomi-cho, Tonami City, Toyama 939-1395 Japan; 3 Department of Rehabilitation Medicine, Toyama Prefectural Rehabilitation Hospital and Support Center for Children with Disabilities, 36 Shimoiino-machi, Toyama, 939-1395 Japan; 4 Department of Rehabilitation Medicine, Kanazawa University Hospital, 13-1 Takara-machi, Kanazawa City, Ishikawa 920-8641 Japan; 5 Department of Orthopaedic Surgery, Graduate School of Medicine, Kanazawa University, 13-1 Takara-machi, Kanazawa City, Ishikawa 920-8641 Japan

**Keywords:** Baclofen, Intrathecal catheter, Lumbar spinal stenosis, Decompression

## Abstract

**Background:**

Intrathecal baclofen therapy can substantially improve symptoms in most patients with severe spasticity due to traumatic spinal cord injury, multiple sclerosis, or cerebral paresis. To the best of our knowledge, decompression surgeries at the intrathecal catheter insertion site in patients with a preexisting intrathecal pump for drug delivery have not been reported.

**Case presentation:**

We report the case of a 61-year-old Japanese man with lumbar spinal stenosis who underwent intrathecal baclofen therapy. We performed decompression for lumbar spinal stenosis at the intrathecal catheter insertion site during intrathecal baclofen therapy. The yellow ligament was removed by partial resection of the lamina under a microscope to avoid damage to the intrathecal catheter. The dura mater was distended. No obvious cerebrospinal fluid leakage was observed. Postoperatively, lumbar spinal stenosis symptoms improved, and spasticity remained well controlled with intrathecal baclofen therapy.

**Conclusions:**

This is the first reported case of lumbar spinal stenosis decompression at an intrathecal catheter insertion site during intrathecal baclofen therapy. Preoperative preparation is necessary, as the intrathecal catheter may be replaced during surgery. We performed surgery without removing or replacing the intrathecal catheter, taking care not to damage the spinal cord by migrating the intrathecal catheter.

## Background

To the best of our knowledge, decompression surgeries at the intrathecal catheter insertion site in patients with a preexisting intrathecal pump for drug delivery have not been reported. We report a case of decompression for lumbar spinal stenosis (LSS) at the intrathecal catheter insertion site during intrathecal baclofen (ITB) therapy.

## Case presentation

**Fig. 1:**
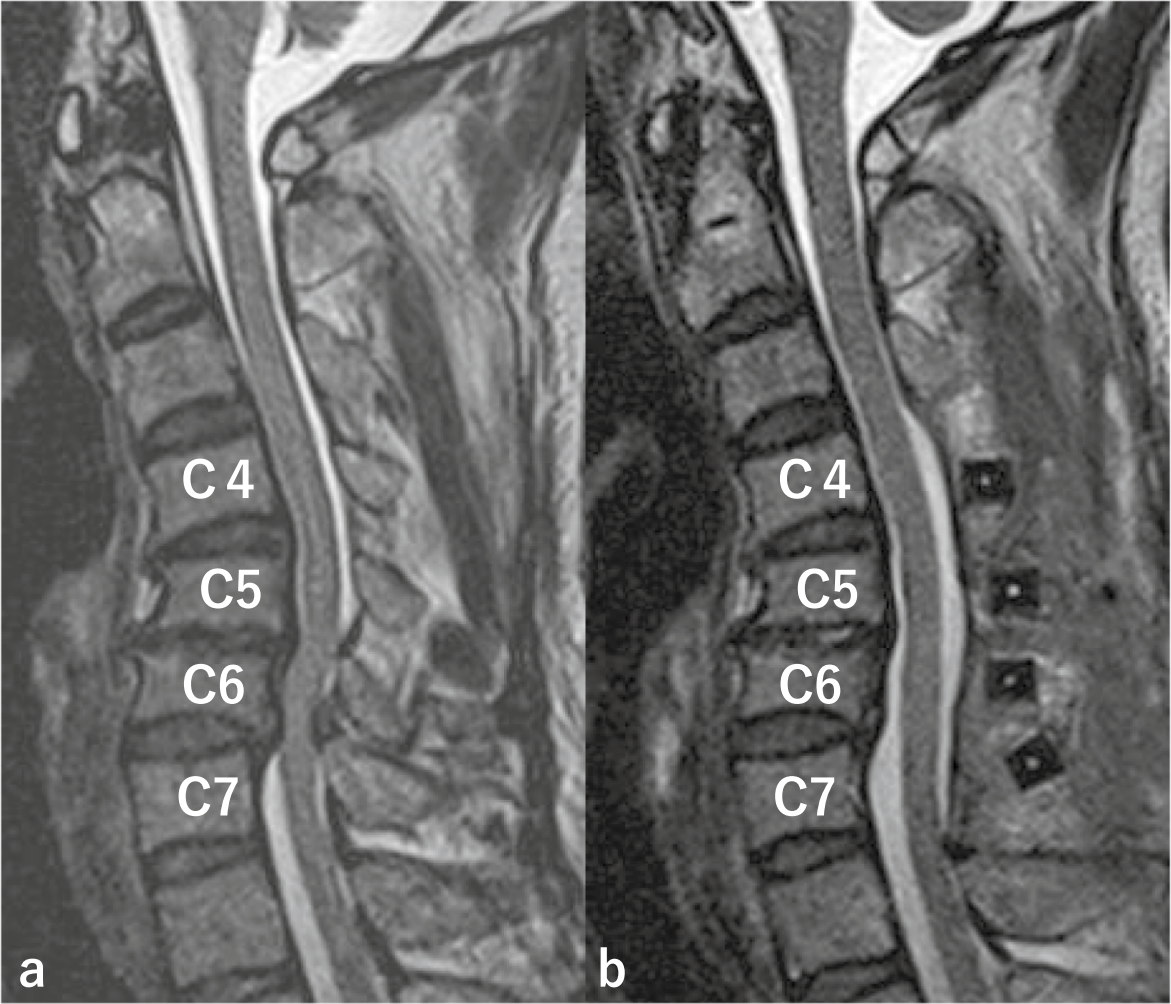
Preoperative magnetic resonance imaging (MRI) and MRI after laminoplasty. Preoperative MRI shows spinal canal stenosis at C5/6 and C6/7, and MRI after laminoplasty reveals decompression of the spinal cord. **a** Preoperative MRI, **b** MRI after laminoplasty

**Fig. 2:**
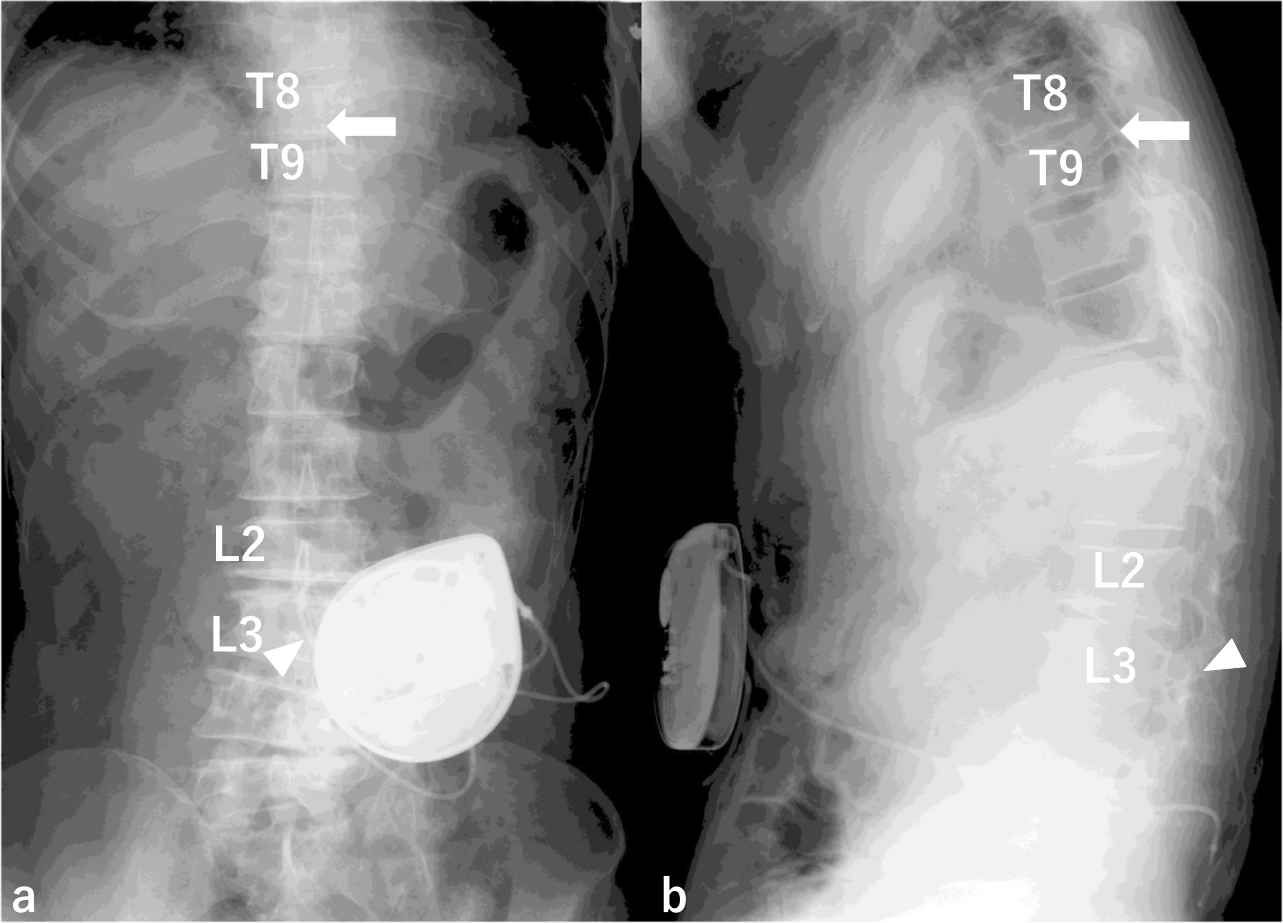
Intrathecal baclofen (ITB) pump implantation. ITB pump implantation was performed by inserting the intrathecal catheter through L2/3 interspace (white solid triangle) to T8/9 level (white solid arrow). **a** AP view **b** Lateral view

**Fig. 3:**
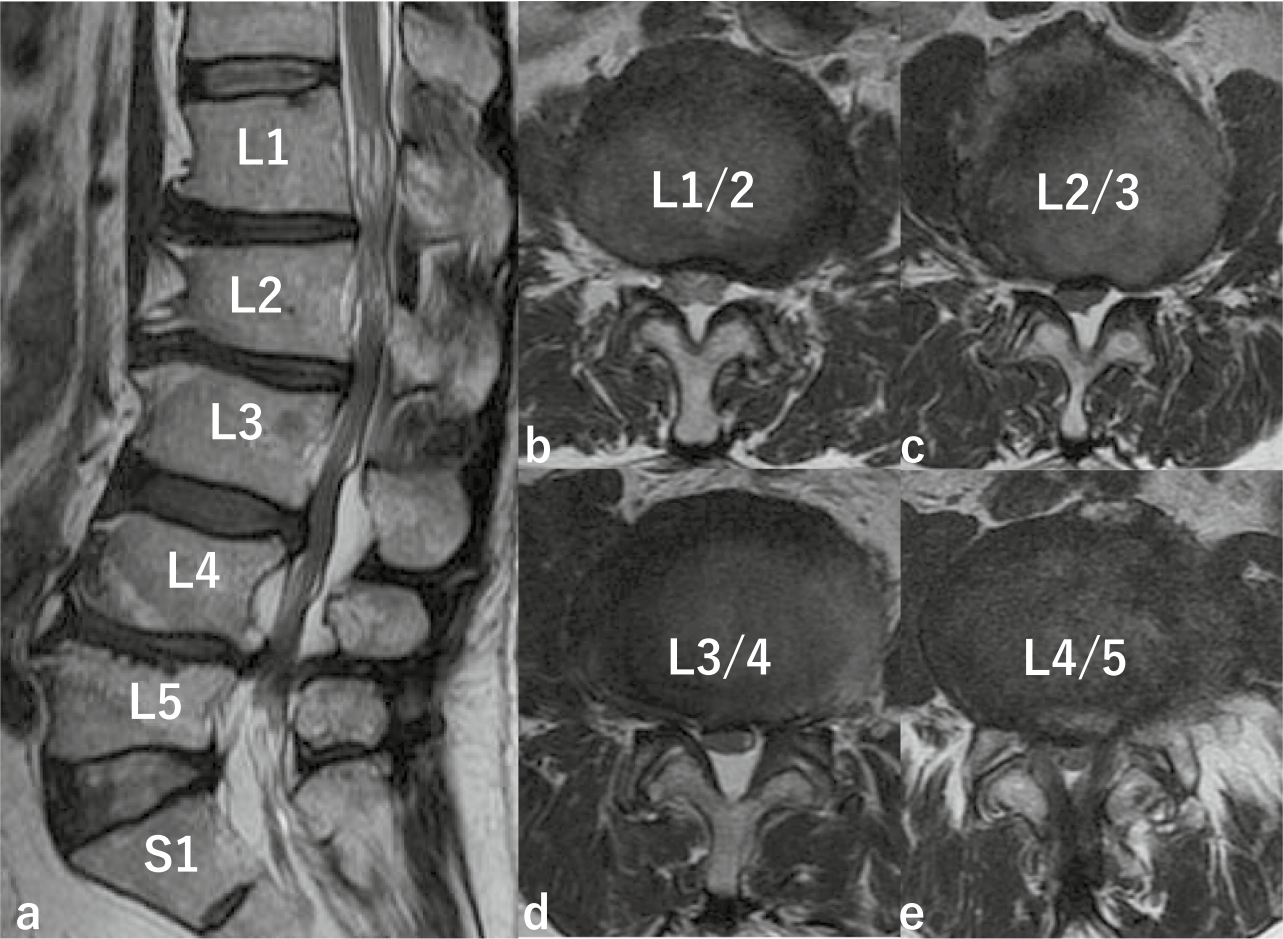
MRI of the lumbar spine. MRI shows multiple lumbar spinal stenosis (LSS) on T2-weighted imaging (**a** sagittal; **b** L1/2 axial; **c** L2/3 axial; **d** L3/4 axial; **e** L4/5 axial)

A 61-year-old Japanese man had a cervical spinal cord injury approximately 2 years previously, and underwent cervical laminoplasty from C4 to C7 at a different hospital. Preoperative magnetic resonance imaging (MRI) revealed spinal canal stenosis at C5/6 and C6/7, and MRI after laminoplasty revealed decompression of the spinal cord (Fig. 1). He was referred to our hospital 6 months after surgery because of progressively worsening spasticity of the lower limbs. We performed ITB pump (SynchroMed II, Medtronic, Inc., Minneapolis, MN, USA) implantation by inserting an intrathecal catheter through the L2/3 interlaminar space to the T8/9 level (Fig. 2). After surgery, his spastic gait improved and progressed well; however, 1 year after surgery, intermittent claudication was observed, making it difficult for the patient to walk long distances. Physical examination revealed numbness and mild muscle weakness in both lower extremities. No signs of spinal tension were observed. MRI of the lumbar spine revealed multiple LSS at L1/2, L2/3, L3/4, and L4/5 (Fig. 3). Based on these findings, we planned to decompress all stenotic areas. We also considered the possibility that the intrathecal catheter inserted through the L2/3 interlaminar space would need to be removed in preparation for surgery.

## Results

**Fig. 4:**
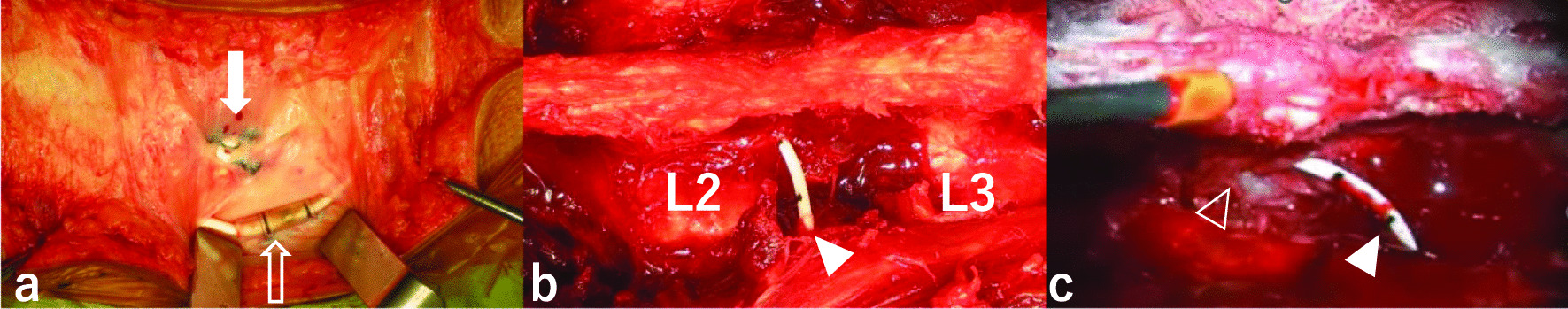
Intraoperative findings. The connector pin and sleeve (white outline arrow) connecting the intrathecal catheter’s pump segment to the spinal segment and anchor (white solid arrow) securing the spinal segment of the intrathecal catheter. The spinal segment of the intrathecal catheter (white solid triangle) through L2/3 interlaminar space. The dura mater (white outline triangle) is distended. **a** superficial layer **b** deep layer **c** post decompression

Electrocautery was not used due to the risk of impaired pump operability. As we deployed the subcutaneous tissue, we found the connector pin and sleeve connecting the pump segment of the intrathecal catheter to the spinal segment and the anchor securing the spinal segment of the intrathecal catheter. Once we opened the lumbar lamina, we observed the spinal segment of the intrathecal catheter inserted through the L2/3 interlaminar space. The intrathecal catheter and L2 lamina proximal to the intrathecal catheter was confirmed under a microscope. Partial resection of the L2 lamina was performed with a high-speed diamond-tipped burr with a diameter of 4 mm under a microscope. The intrathecal catheter was then retracted with a nerve root retractor, and resection of the ligamentum flavum and partial resection of L3 lamina were performed with the Kerrison rongeur under a microscope, so as not to damage the intrathecal catheter. When retracting the intrathecal catheter with a nerve root retractor, the retraction was kept to a minimum so as not to damage the spinal cord at the tip of the intrathecal catheter. The dura mater was distended, and no obvious cerebrospinal fluid leakage was observed (Fig. 4).

**Fig. 5:**
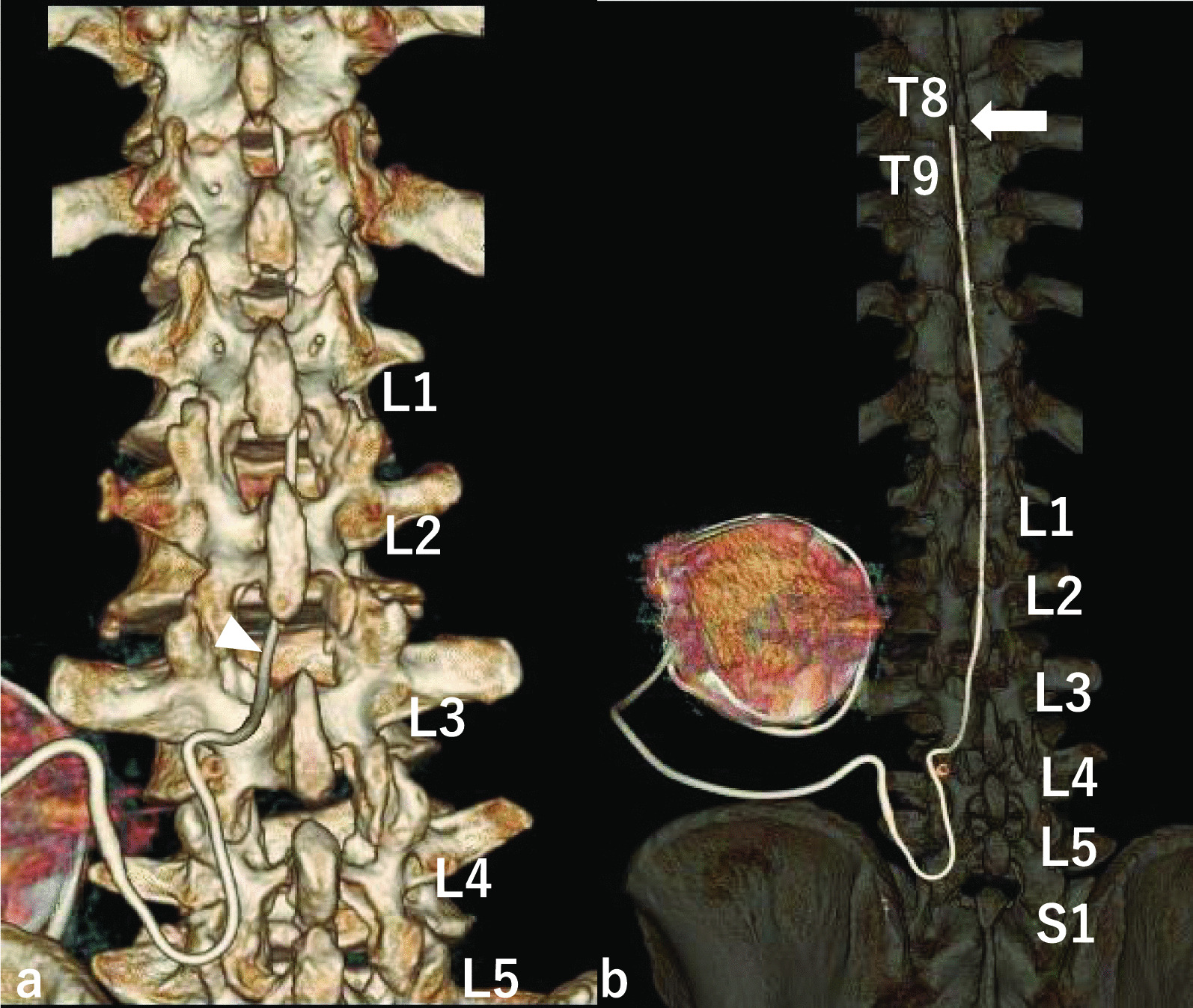
Postoperative three-dimensional computed tomography (3D-CT) scan. Postoperative 3D-CT scan confirms that the intrathecal catheter was inserted through the L2/3 interlaminar space to the original position T8/9 level without damaging the intrathecal catheter, and lumbar spinal stenosis (LSS) was decompressed. White solid arrow: the tip of the intrathecal catheter. White solid triangle: the insertion site of the intrathecal catheter. **a** 3D-CT **b** 3D-CT Easier to see the intrathecal catheter

**Fig. 6:**
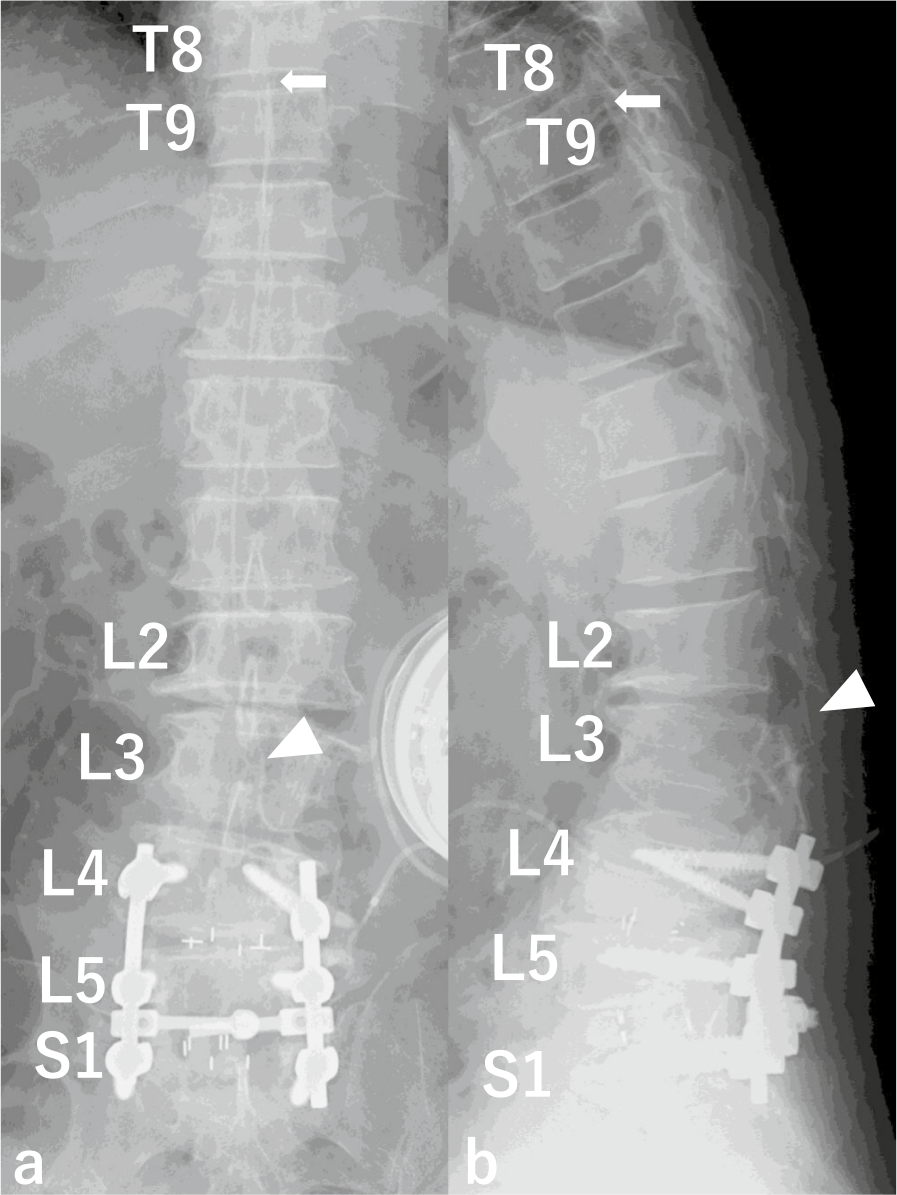
Postoperative X-ray. The intrathecal catheter is inserted through the L2/3 interlaminar space to the original position T8/9 level. White solid triangle: the insertion site of the intrathecal catheter. **a** AP view **b** Lateral view

Postoperative three-dimensional computed tomography confirmed that the intrathecal catheter was inserted through the L2/3 interlaminar space to the original position at the T8/9 level without any damage to the intrathecal catheter and the LSS was decompressed (Fig. 5). Postoperatively, the patient’s gait was good, and although his spasticity persisted, he progressed well with ITB therapy. The pain in the left lower extremity recurred 8 months after surgery. We diagnosed the patient with neurological symptoms of lumbar foraminal stenosis with associated scoliosis. Additional posterior lumbar interbody fusion (PLIF) was performed at L4/5 and L5/S. Deployment was performed as previously described. The spinal segment of the intrathecal catheter could not be identified because the surgical site was distal to the intrathecal catheter insertion site. X-ray examination after PLIF revealed that the intrathecal catheter was inserted through the L2/3 interlaminar space to the original T8/9 level (Fig. 6). Additional lumbar fusion surgery improved the gait, and 9 years and 5 months after lumbar fusion surgery spasticity continued to be well controlled with ITB therapy. We performed both operations without removing or replacing the intrathecal catheter, taking care not to damage the intrathecal catheter or spinal cord.

## Discussion and conclusions

ITB effectively improves spasticity in patients whose spasticity is not sufficiently managed by oral baclofen or other oral antispastic medications. Baclofen withdrawal syndrome is a potentially life-threatening complication [1–8].

To the best of our knowledge, decompression surgeries at the intrathecal catheter insertion site in patients with a preexisting intrathecal pump for drug delivery have not been reported. There are only two reports of posterior spinal fusion in which baclofen was not delivered despite a normal functioning pump [9, 10]. Alden *et al*. [9] reported a case of posterior spinal fusion for scoliosis in which baclofen was not delivered despite the use of a normal functioning pump. The report offered no details concerning the type of surgery or possible cause of intrathecal catheter malfunction. Fernandes *et al*. [10] reported a case of posterior spinal fusion for neuromuscular scoliosis that developed ITB withdrawal syndrome, likely caused by a small nick created in the intrathecal catheter tubing during dissection. The authors described three available surgical methods to prevent this complication. First, the intrathecal catheter was removed during the exposure and reintroduced at the end of the procedure. Second, intrathecal catheter preservation can be attempted through delicate dissection and isolation. Third, the intrathecal catheter was sectioned and reattached at the end of the procedure using a connector. The first option is related to low-pressure headaches secondary to presumed cerebrospinal fluid leakage. The second option has potential complications during the procedure: catheter injury may occur at various points, and the intrathecal catheter can be pulled out to a level distal to the intended level or from the spinal canal itself. The third option must be coordinated with the neurosurgical service to ensure that the pump catheter system functions properly afterwards.

We report the first case of decompression for LSS at an intrathecal catheter insertion site during ITB therapy. Preoperative preparation is necessary as the intrathecal catheter may be replaced during surgery. Care was taken not to damage the intrathecal catheter or spinal cord, and treatment was possible without removing or replacing the intrathecal catheter. Surgeons must be aware of ITB withdrawal syndrome and develop strategies to prevent interruption of baclofen delivery.

This is the first report of decompression of LSS at an intrathecal catheter insertion site during ITB therapy. Preoperative preparation is necessary as the intrathecal catheter may be replaced during surgery. Care was taken not to damage the intrathecal catheter or spinal cord, and treatment was possible without removing or replacing the intrathecal catheter.

## Data Availability

Medical imaging data will not be shared because it is not fully anonymous.

## Abbreviations

ITB: Intrathecal baclofen
LSS: Lumbar spinal stenosis
MRI: Magnetic resonance imaging
PLIF: Posterior lumbar interbody fusion
